# Supplementary material for: Knowledge and practices of birth preparedness and complication readiness among pregnant women in Eastern Province, Zambia: A qualitative study
Source: PLOS Glob Public Health. 2025 Jul 24;5(7):e0004975. doi: 10.1371/journal.pgph.0004975 (PMC12289000; doi:10.1371/journal.pgph.0004975)
Supplement: Attachment — Submitted filename: Review comments_Birth Preparedness.pdf [file pgph.0004975.s001.pdf]

Manuscript: PGPH-D-25-00507

Title: Knowledge and Practices of Birth Preparedness and Complication Readiness among Pregnant Women in Eastern Province, Zambia: A Qualitative Study

Review comments

To begin with, thank you very much for providing the opportunity to review this manuscript. In addition, I will list out all the important comments to be incorporated within the manuscript for a better research manuscript.

Abstract

1. Page: 2; line 20-39: The abstract of the manuscript should breakdown in to components such as introduction, objective, methodology, result, conclusion and recommendations for easily understand the research overall outputs.
2. Page: 4; line 68-69: Use either of Vancouver or Harvard reference style. Don't use both references throughout the manuscript.
3. Page: 4; line 68-69: ... only a small proportion of women were prepared for birth and its complications. How much proportion? It should be figurative in percentage.
4. Page: 4; line 74-75: How much you are sure that no documented studies about BP/CR in Zambia? Sample studies are here. https://dspace.unza.zm/server/api/core/bitstreams/f6f5b3f7-f944-4d87-9b1f-2c1bf8385fa5/content

   Methodology

5. Page: 5; line 89-90: Zambia's MMR is 278 per 100,000 live births and a stillbirth rate of 15.9 per 1000 births [3]. Avoid duplication of explanations. In addition, no need of adding this sentence here at all. Therefore, remove it.
6. Page: 5; line 90-93: The authors are explaining the significance of conducting this study rather than explaining the study setting more elaborately. Please focus your explanations more on about the study setting such as what is eastern provinces? How many districts does eastern provinces have? How many is the population and pregnant women available during the study period? How many health facilities are available with in the eastern provinces and selected districts?
7. Page: 5; line 99: What does a subsequent ANC contacts mean? How many ANC visits did a pregnant women had to be participated in your study?

Result

8. Page 17: line 295-297: Did the participant (R8: 297 FGD Tafelansoni RHC) was not a pregnant woman attending ANC visit? She is talked about giving her blood to her mum but the question was who could donate blood to them. It is not clear.
9. Page 17: line 309-310: I think the sentence written was not explained what intended to explained about the health seeking behavior. Please rewrite it.
10. Page 21: line 382: How did you measure that the knowledge of complication readiness among the ANC attendees was low?

Conclusion

11. Page 25: line 470-475: Your conclusions must be based on your findings.

Recommendations

12. I can't find the authors recommendations based on the article findings to relevant bodies. It should be included.

Ethical approval

13. The ethical issues are explained two times. Once in page 7: line 133-139 and the second is in page; 26 line 492-500. Therefore, it should be explained once after the

Overall comment

This study is well-conducted, and the researchers are commended for their work thorough evaluating Knowledge and Practices of Birth Preparedness and Complication Readiness among Pregnant Women in Eastern Province, Zambia: A Qualitative Study. The study is effectively structured, with a clear identification of the impact of Knowledge and Practices of Birth Preparedness and Complication Readiness among Pregnant Women in Eastern Province, Zambia for public health interventions. However, to enhance its credibility and ensure clear communication of the findings, the researchers should address the minor comments and suggestions provided above. Once these revisions are made, the study will be ready for publication and will provide valuable insights into decrease maternal complications and morbidity.
