# Supplementary material for: Knowledge and practices of birth preparedness and complication readiness among pregnant women in Eastern Province, Zambia: A qualitative study
Source: PLOS Glob Public Health. 2025 Jul 24;5(7):e0004975. doi: 10.1371/journal.pgph.0004975 (PMC12289000; doi:10.1371/journal.pgph.0004975)
Supplement: Attachment — Submitted filename: Response to Reviewers.pdf [file pgph.0004975.s003.pdf]

The University of Zambia
School of Public Health
Department of Epidemiology and Biostatistics
Box 32379, Lusaka, Zambia

9th June 2025

The Editor
PLOS Global Public Health

Dear Sir/ Madam,

Ref: Rebuttal to Editors and Reviewers comments for Manuscript Titled "**Knowledge and Practices of Birth Preparedness and Complication Readiness among Pregnant Women in Eastern Province, Zambia: A Qualitative Study**" for publication

We would like to thank you for reviewing our manuscript and noting that it has merit.

I wish to inform the Editor that the manuscript has been revised in line with the Editors and Reviewers comments. The table below addresses the points raised during the review process.

We look forward to receiving a favourable response to our revised manuscript.

Kind regards,

Dr. Muyereka Nyirenda

Response to PLOS One Reviewer's Comments based on revised manuscript_ PGPH-D-25-00507

S/N	Query	Response
	**Editors and Reviewers Comments**	
	**Abstract**	
1.	Page: 2; line 20-39: The abstract of the manuscript should breakdown in to components such as introduction, objective, methodology, result, conclusion and recommendations for easily understand the research overall outputs.	Abstract broken into background, methods, results and conclusions. **Page 2, lines 21-40**
2.	Page: 4; line 68-69: Use either of Vancouver or Harvard reference style. Don't use both references throughout the manuscript.	

		Correction made, Vancouver in-text citation maintained. **Page 4, line 68** and **page 25, line 467**
3.	Page: 4; line 68-69: ... only a small proportion of women were prepared for birth and its complications. How much proportion? It should be figurative in percentage.	These were responses from participants in focus group discussions (FGDs) hence proportions or percentages may not be used.
4.	Page: 4; line 74-75: How much you are sure that no documented studies about BP/CR in Zambia? Sample studies are here.	The sampled study took a quantitative approach. This study took a qualitative approach.
	**Methodology**	
5.	Page: 5; line 89-90: Zambia's MMR is 278 per 100,000 live births and a stillbirth rate of 15.9 per 1000 births [3]. Avoid duplication of explanations. In addition, no need of adding this sentence here at all. Therefore, remove it.	This sentence has been removed from methodology section
6.	Page: 5; line 90-93: The authors are explaining the significance of conducting this study rather than explaining the study setting more elaborately. Please focus your explanations more on about the study setting such as what is eastern provinces? How many districts does eastern provinces have? How many is the population and pregnant women available during the study period? How many health facilities are available with in the eastern provinces and selected districts?	The study setting has been revised, making it more elaborate. **Page 5, lines 89-93**.
7.	Page: 5; line 99: What does a subsequent ANC contacts mean? How many ANC visits did a pregnant women had to be participated in your study?	Pregnant women who had attended a minimum of two ANC visits. Meaning that they had attended at least two health education sessions. **Page 5, line 100.**
	**Results**	
8.	Page 17: line 295-297: Did the participant (R8: 297 FGD Tafelansoni RHC) was not a pregnant woman attending ANC visit? She is talked about giving her blood to her mum but the question was who could donate blood to them. It is not clear.	The statement has been clarified. The participant did not donate blood when she was pregnant. Pregnant women do not donate blood in our setting. **Page 17, lines 306-307**
9.	Page 17: line 309-310: I think the sentence written was not explained what intended to explained about the health seeking behavior. Please rewrite it.	The sentence has been re-written for clarity. **Page 18, lines 319-320.**

10.	Page 21: line 382: How did you measure that the knowledge of complication readiness among the ANC attendees was low?	This being a qualitative study, the measure was based on the participant response during focus group discussions.
	**Conclusion**	
11.	Page 25: line 470-475: Your conclusions must be based on your findings.	The conclusion has been revised to better reflect the study findings. **Page 26, lines 484-494**.
	**Recommendations**	
12.	I can't find the authors recommendations based on the article findings to relevant bodies. It should be included.	A paragraph on recommendations has been inserted. **Pages 26 and 27, lines 495-505.**
	**Ethical approval**	
13.	The ethical issues are explained two times. Once in page 7: line 133-139 and the second is in page; 26 line 492-500. Therefore, it should be explained once after the	The paragraph on ethical consideration has been revised. **Page 7 and 8, lines 139-149**. The second one that was on page 26 has been removed.
